# Lactate promotes PGE2 synthesis and gluconeogenesis in monocytes to benefit the growth of inflammation-associated colorectal tumor

**DOI:** 10.18632/oncotarget.3838

**Published:** 2015-04-15

**Authors:** Libin Wei, Yuxin Zhou, Jing Yao, Chen Qiao, Ting Ni, Ruichen Guo, Qinglong Guo, Na Lu

**Affiliations:** ^1^ State Key Laboratory of Natural Medicines, Jiangsu Key Laboratory of Carcinogenesis and Intervention, China Pharmaceutical University, Nanjing, The People's Republic of China; ^2^ Xi'an Middle School of Shanxi Province, Xi'an, The People's Republic of China

**Keywords:** lactate, HIF-1α, gluconeogenesis, inflammation, microenvironment

## Abstract

Reprogramming energy metabolism, such as enhanced glycolysis, is an Achilles' heel in cancer treatment. Most studies have been performed on isolated cancer cells. Here, we studied the energy-transfer mechanism in inflammatory tumor microenvironment. We found that human THP-1 monocytes took up lactate secreted from tumor cells through monocarboxylate transporter 1. In THP-1 monocytes, the oxidation product of lactate, pyruvate competed with the substrate of proline hydroxylase and inhibited its activity, resulting in the stabilization of HIF-1α under normoxia. Mechanistically, activated hypoxia-inducible factor 1-α in THP-1 monocytes promoted the transcriptions of prostaglandin-endoperoxide synthase 2 and phosphoenolpyruvate carboxykinase, which were the key enzyme of prostaglandin E2 synthesis and gluconeogenesis, respectively, and promote the growth of human colon cancer HCT116 cells. Interestingly, lactate could not accelerate the growth of colon cancer directly *in vivo*. Instead, the human monocytic cells affected by lactate would play critical roles to ‘feed’ the colon cancer cells. Thus, recycling of lactate for glucose regeneration was reported in cancer metabolism. The anabolic metabolism of monocytes in inflammatory tumor microenvironment may be a critical event during tumor development, allowing accelerated tumor growth.

## INTRODUCTION

Warburg effect is a common feature of cancer cells. In over 70% of all human cancers worldwide, the glycolysis-related genes are overexpressed [1]. Thus, tremendous amounts of researches in the field of tumor glycolysis and lactate metabolism are being in progress. To meet the need of rapid growth, cancer cells increases glucose uptake and accumulates lactate. This phenomenon clearly indicates that lactate is not a surrogate of tumor hypoxia. More than a metabolic waste, the lactate anion is known to participate to cancer aggressiveness, including metastasis, radio resistance, angiogenesis and immune escape of tumor cells [2-5]. These findings suggest that lactate is a metabolic key player as well as a core signaling molecular in cancer.

Lactate is an anion and therefore requires transporters to efficiently cross cell membranes. This function is predominantly exerted by monocarboxylate transporter (MCT) [6]. The transport of lactate is important for tumors, not only to prevent cellular acidification through exporting lactic acid, but also to sustain growth and metastasis through importing lactate [7]. Of the MCT families, MCT1 and MCT4 play predominant roles in the majority of cancer cells. There is a preferential expression of MCT1 in normoxic cells with oxidative potential [8], whereas MCT4 expression is predominant in glycolytic/hypoxic cells [9]. Lactate is primarily exported by MCT4 from glycolytic tumor cells or stromal cells [10], whereas is taken up by MCT1 to participate carbohydrate metabolism.

Previous studies concerning glycolytic metabolism dysfunction gave more attentions on the biochemical regulation and certain factors in isolated tumor cells and tumor cell lines, and ignored recycled nutrients between cells in tumor microenvironment. Tumor microenvironment, which contains stromal fibroblasts, inflammatory cells, vascular endothelial cells and so on, is critical for the initiation and progression of cancer. Currently, the team of Prof. Lisanti reported a new energy-transfer mechanism “Two-Compartment Tumor Metabolism” between cancer cells and cancer associated fibroblasts to reflect that the production, consumption and recycle of nutrients [11, 12]. Inflammation is the indispensable part of tumor microenvironment, and works at all stages of tumorigenesis. Production of tumor-promoting cytokines by immune/inflammatory cells, such as prostaglandin E2 (PEG2), results in the activation of transcription factors and induces expression of genes promoting cancer development [13]. As above described, though some studies involving in the recycle metabolism of cancer associated fibroblasts have been conducted [11], rare crosstalk was investigated between the cancer glycolytic metabolism and tumor inflammatory microenvironment.

Here, we explored energy-transfer mechanism of human monocytic cells in tumor inflammatory microenvironment. THP-1 monocytes were one of most widely used cell lines to investigate the function and regulation of monocytes and macrophages, and an important tool to mimic the inflammatory cells in tumor microenvironment. Interestingly, our experimental evidence indicates the “Two-Compartment Tumor Metabolism” occurred THP-1 monocytes and colon cancer cells. We found lactate secreted by cancer cells promote the synthesis of PGE2 of THP-1 monocytes through stabilizing hypoxia-inducible factor 1α (HIF-1α) in normoxia. HIF-1α is a critical oncogene and transcription factor involving in the metabolic switch from oxidative phosphorylation (OXPHOS) toward an altered glycolysis [14]. HIF-1α is also capable of modulating the transcription of prostaglandin-endoperoxide synthase 2 (COX2), which is the key enzyme for PGE2 synthesis [15, 16]. Usually, HIF-1α is controlled by cellular oxygen concentrations via prolyl hydroxylases (PHDs) and the von Hippel–Lindau (vHL) complex, and is easily degraded in normoxia. However, recent studies demonstrate that HIF-1α is stabilized even under normoxic conditions by the products of glycolysis, lactate and pyruvate [5, 17]. Therefore, lactate from cancer cells functions as an onco-metabolite, stabilizing HIF-1α and stimulating mitochondrial biogenesis in adjacent monocytic cells. In addition to oxidation by lactate dehydrogenase (LDH), lactate became the raw material and engaged in gluconeogenesis in THP-1 monocytes. The transcriptional upregulation of phosphoenolpyruvate carboxykinase (PEPCK) by HIF-1α was responsible for the surge of lactate to gluconeogenesis.

In this context, human monocytic cells recycle lactate to glucose, serving as a high-energy mitochondrial “fuel” for cancer cells growth. The metabolic feedback loop in tumor inflammatory microenvironment drives tumor-inflammation co-evolution. The deep-going studies of the metabolic regulation in tumor inflammatory microenvironment will provide fresh insight into the cancer metabolism, and help us finding the new targets for cancer therapies.

## RESULTS

### THP-1 monocytes co-cultured with colorectal tumor HCT116 cells exhibits active gluconeogenesis-like character

In 2010, a novel theory named “Reversed Warburg effect” challenge the millstone of cancer biology studying, which supported the idea of stromal-epithelial metabolic coupling in the tumor microenvironment. Therefore, we suspected whether there was a similar “Two-Compartment Tumor Metabolism” model in the inflammatory tumor microenvironment.

**Figure 1:**
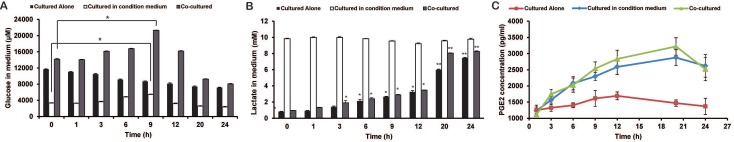

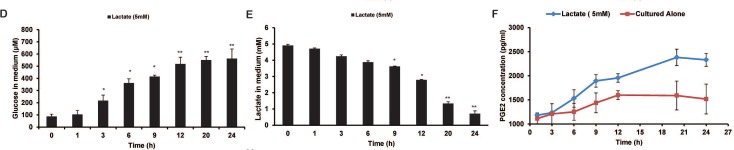

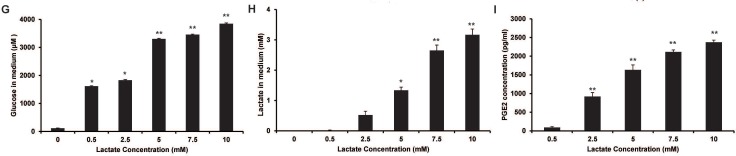
The influence of lactate on glucose metabolism and PGE2 secretion of THP-1 monocytes **A-C.** THP-1 monocytes were stimulated with the condition medium (CM) of HCT116 cells, or co-cultured with HCT116 cells for 24 h. The culture supernatant of different time points were selected. The quantities of glucose **A.**, lactate **B.**, and PGE2 **C.** in the supernatant were measured by Amplex Red assay kit, Lactic Acid production Detection kit, and PGE2 ELISA kit, respectively. **D-F.** After THP-1cells were stimulated with 5 mM lactate, the quantities of glucose **D.**, lactate **E.**, and PGE2 **E.** in the supernatant were measured. **G-I.** The quantities of glucose **G.**, lactate **H.**, and PGE2 **I.** in the supernatant of THP-1 monocytes upon different concentrations of lactate for 12 h were assayed. Bars, SD; *p < 0.05 or **p < 0.01 versus untreated controls.

In order to investigate the glycolysis metabolism of inflammatory cells in the tumor microenvironment, we cultured human colorectal cancer HCT116 cells for 48 h and used the condition medium (CM) to stimulate human THP-1 monocytes for various times. Surprisingly, the glucose in CM did not decrease until THP-1 was stimulated for 12 h (Figure 1A); and the lactate in CM was little changed (Figure 1B). This phenomenon is seemed that THP-1 monocytes used the lactate in CM for regeneration of glucose instead of energy production, which exactly is similar to the process of gluconeogenesis. To further identify the speculation that THP-1 monocytes came into gluconeogenesis in the inflammatory tumor microenvironment, we co-cultured THP-1 monocytes with HCT116 cells by using Milli hang culture well. As a result, though the secreted lactate increased gently within 12 h (Figure 1A), the glucose in the co-cultured medium still appeared an ascending trend for 12 h co-culture, and declined later (Figure 1B). We even contacted HCT116 cells with THP-1 monocytes, and obtained the consistent result in the change of glucose amount (Figure S1A). Besides in human colon cancer HCT116 cells, we also tested this model in other human cancer cell lines, including human colon cancer HT29 cells, Caco2 cells, human hepatoma HepG2 cells, and human breast cancer MDA-MB-231 cells. The trends of lactate and glucose in the models of other cancer cell lines were the same as that of HCT116 cells (Figure S2A and S2B). Especially, glucose in both CM and co-cultured medium for THP-1 monocytes appeared ascending trend first, and declined later (Figure S2B). All the results suggested that the gluconeogenesis of THP-1 monocytes could be triggered by cancer cells in the inflammatory tumor microenvironment.

### The generation of PGE2 of THP-1 monocytes was promoted by colorectal tumor HCT116 cells

PGE2 is a bioactive lipid that can elicit a wide range of biological effects associated with inflammation and cancer. Subsequently, the influences of cancer cells on PGE2 secretion of inflammatory cells were investigated. We detected the content of PGE2 in the culture medium by ELISA method. Compared with the THP-1 monocytes cultured solely, the release of PGE2 in THP-1 monocytes stimulated with culture supernatant liquid of HCT116, or co-cultured with HCT116 cells using hang culture well (Figure 1C), or contact co-cultured (Figure S1B), were all increased. As well, the release of PGE2 in THP-1 monocytes stimulated by other cancer cell lines were increased (Figure S2C). These results demonstrated that cancer cells promoted the generation of PGE2 of THP-1 monocytes.

### Lactate was taken up through MCT1 and promoted the gluconeogenesis and PGE2 generation of THP-1 monocytes

Based above phenomenon, we wondered what the signaling molecules released by cancer cells, stimulated the gluconeogenesis and PGE2 generation of THP-1 monocytes. Considering all the three culture models of THP-1 monocytes (including simulated with CM, co-cultured in hang culture well, and contact co-cultured) resulted in glycogenesis and increased PGE2 secretion, we exclude the influence of cell contact and pointed to some certain molecules secreted by cancer cells. After HCT 116 cells were cultured for 48 h, the culture supernatant liquid accumulated lactate and contained little glucose (Figure S1C and S1D). Lactate is the byproduct of “Warburg effect” as well as the raw material gluconeogenesis. Therefore, we picked out lactate to explore its functions from the numerous of factors in inflammatory tumor microenvironment.

When HCT116 cells were cultured alone for 48 h, the concentration of lactate reached nearly 5 mM (Figure S1C). Therefore, for mimic the acidic environment, we added lactate solution to the glucose-free culture medium and used 5 mM as an initial reference concentration to stimulate THP-1 monocytes. As a result, the lactate in the medium was decreased, and glucose generation and PGE2 release were both increased along with the increased concentration or time of lactate stimulation (Figure 1D to 1I, and Figure S1B). Instead, little influence on cell growth was observed (Figure S1E, S1F and S1G).

**Figure 2:**
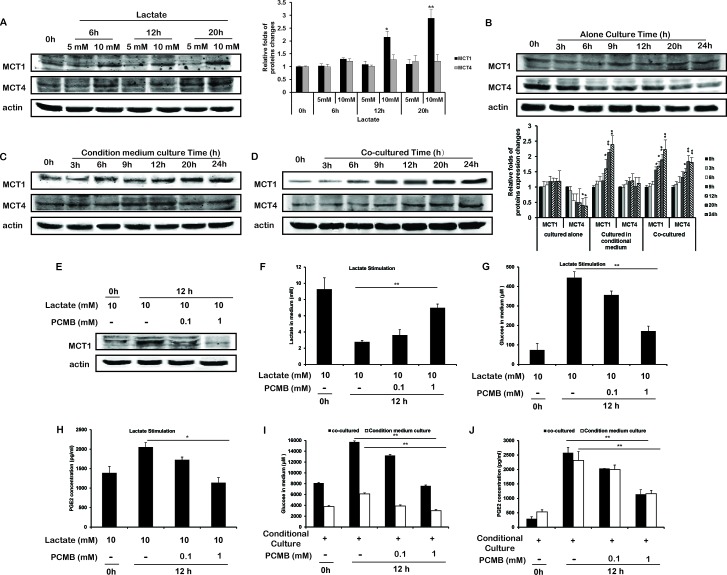
Lactate upregulated the expression of MCT1 in THP-1 monocytes **A-D.** The protein expressions of MCT1 and MCT4 in THP-1 monocytes stimulated with lactate **A.**, cultured alone **B.**, cultured in CM **C.**, or co-cultured with HCT116 cells **D.** were measured. Protein bands were quantified. **E-H.** THP-1 monocytes were treated with 10 mM lactate without or in the presence of PCMB for 12 h. MCT1 protein expression **E.**, and the quantities of lactate **F.**, glucose **G.**, and PGE2 **H.** in the supernatant were measured. **I, J.** THP-1 monocytes were stimulated with the condition medium and/or PCMB for 12 h. Glucose **I.** and PGE2 **J.** in the supernatant were measured. Bars, SD; *p < 0.05 or **p < 0.01 versus untreated controls.

Among monocarboxylate transports (MCT) family, MCT1 and MCT4 were mainly responsible for the export and import of lactate, respectively. Upon the stimulation of lactate, MCT1 protein expression of THP-1 monocytes was increased, but MCT4 not (Figure 2A). Moreover, stimulation of THP-1 monocytes with CM of HCT116 cells(Figure 2C), or co-cultured with HCT116 cells using hang culture well (Figure 2D), resulted in the upregulation of MCT1 protein expression as well. In the models based on other cancer cells, MCT1 protein expressions of THP-1 monocytes were enhanced by stimulated of CM or co-cultured with cancer cells (Figure S3).

To further verify the effects of lactate and MCT1, we used 4-(Chloromercuric) benzoic acid (PCMB, MCT1 inhibitor [18]) to inhibit the lactate uptake (Figure 2E and 2F). PCMB inhibited the lactate-induced gluconeogenesis and PGE2 production in THP-1 monocytes (Figure 2G and 2H). The glucose generation and PGE2 secretion in THP-1 monocytes stimulated with CM, were also inhibited by PCMB (Figure 2I and 2J).

The above results showed that lactate was an important inducer of the gluconeogenesis and PGE2 generation in THP-1 monocytes in the tumor microenvironment. MCT1 was responsible for the lactate uptake, and upregulated by lactate stimulation in return.

### Lactate upregulated the transcription of PEPCK and COX2 in THP-1 monocytes both *in vitro* and *in vivo*

**Figure 3:**
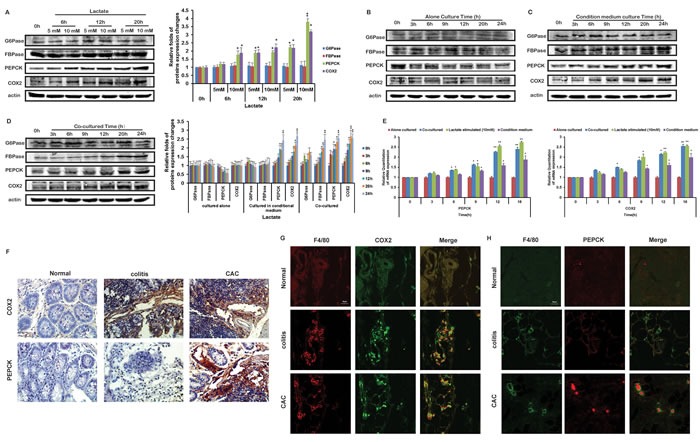
Lactate influenced the protein levels of COX2 and PEPCK in THP-1 monocytes The protein expressions of COX2, PEPCK, FBPase and G6Pase in THP-1 monocytes stimulated with lactate **A.**, cultured alone **B.**, cultured in CM **C.**, or co-cultured with HCT116 cells **D.** were detected by Western blot. Protein bands were quantified. **E.** The mRNA expressions of COX2 and PEPCK in THP-1 monocytes stimulated with lactate, cultured alone, cultured in CM, or co-cultured with HCT116 cells were detected by Realtime PCR, respectively. **F.** The modes of AOM-based colitis-associated cancer (CAC) and acute colitis by using C57 mice were constructed. Expressions of COX2 and PEPCK proteins were detected by IHC. **G, H.** Expression of COX2 **G.** and PEPCK **H.** proteins were detected by IF. The staining of F4/80 was used to distinguish monocytes from the other cells in tissue.

From the above results, we inferred that lactate acted as a key regulator of glucose and PGE2 generation in THP-1 monocytes co-cultured with HCT116 cells. Glucose-6-phosphatase (G6Pase), fructose-1,6-bisphosphatase (FBPase) and phosphoenolpyruvate carboxykinase (PEPCK), are the three key rate-limiting enzymes controlling gluconeogenesis. To investigate the further mechanism, we assayed the influence of lactate on these rate-limiting enzymes of gluconeogenesis firstly. Levels of the three enzymes were little changed in THP-1 monocytes cultured alone (Figure 3A and 3E). And 10 mM lactate could increase the protein and gene expressions of PERCK in THP-1 monocytes, instead had little effects on G6Pase and FBPase (Figure 3B and 3E). Under co-cultured and CM culture, the level of PEPCK as well as FBPase of THP-1 monocytes were increased (Figures 3C, 3D and 3E). On the other hand, COX2 is an enzyme to produce PGE2. In the studies the mRNA expression and protein expression of COX2 were both increased in THP-1 monocytes upon the lactate treatment, as well as in co-cultured and CM cultured THP-1 monocytes (Figure 3B to 3E). Beside, expressions of COX2 and PEPCK protein in THP-1 monocytes cultured in CM or with cancer cells were enhanced in the models based on other cancer cells (Figure S3)

To verify our results *in vivo*, we modeled mice bearing acute colitis and colitis-associated cancer (CAC). The immunohistochemical staining demonstrated that both the colitis group and CAC group showed high level of COX2 (Figure 3F). In addition, apparently higher level of PEPCK was only observed in CAC group (Figure 3F). To distinguish the monocytes with colon cancer cells, F4/80 was used to mark monocytes [19]. The tissue immunofluorescence results showed both the colitis group and CAC group showed high expression of COX2 (Figure 3G). However, the high expressions of PEPCK (Figure 3H) were only appeared in CAC group.

### HIF1α of THP-1 monocytes was stabilized by lactate through inactivating PHDs

**Figure 4:**
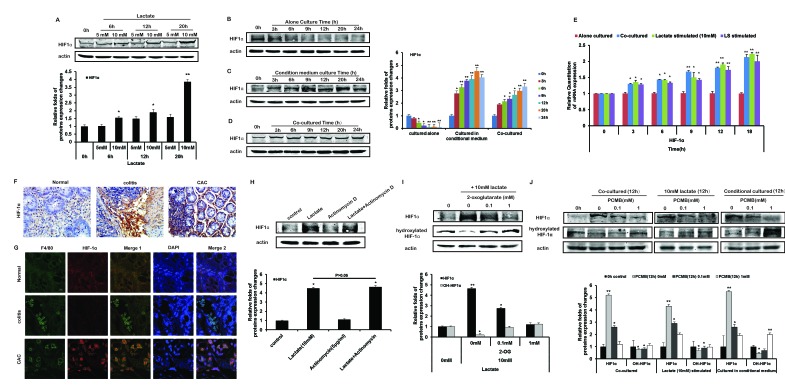
Lactate stabilize HIF-1α of THP-1 monocytes by inhibiting PHD activity under normoxia **A-D.** HIF-1α protein expression in THP-1 monocytes stimulated with lactate **A.**, cultured alone **B.**, cultured in CM **C.**, or co-cultured with HCT116 cells **D.** were detected by Western blot. The actin controls of Figure 4A to 4D were respectively as the same as the control (the same protein band) for total protein in Figure 2A to 2D. Because the actual data were from the same experimental dataset. Protein bands were quantified. **E.** The mRNA expressions of HIF-1α were detected by Realtime PCR. **F.** The modes of AOM-based colitis-associated cancer (CAC) and acute colitis were constructed. Expression of HIF-1α was detected by IHC. **G.** Expression of HIF-1α was detected by IF. The figures of Merge 1 were merged by figures marked with F4/80 and HIF-1α; the figures of Merge 2 were merged Merge 1 with and DAPI staining. **H.** HIF-1α protein expression was detected by Western blot in THP-1 monocytes incubated with 10 mM lactate and/or 5 μg/ml Actinomycin D for 12 h. **I.** HIF-1α and hydroxylated HIF-1α protein expressions were detected by Western blot in THP-1 monocytes incubated with 10 mM lactate and/or increasing concentrations of 2-oxoglutarate for 12 h. **J.** In THP-1 monocytes stimulated with lactate, cultured in CM, or co-cultured with HCT116 cells, with/without increasing concentrations of PCMB for 12 h, the protein expressions of HIF-1α and hydroxylated HIF-1α, were detected by Western blot. Protein bands were quantified. Bars, SD; *p < 0.05 or **p < 0.01 versus untreated controls.

HIF-1α is a key regulator of tumor microenvironment, which regulates the transcription of many factors involving with metabolism, inflammatory response, and so on. HIF-1α was degraded quickly in normoxic environment (Figure 4B). Whereas after THP-1 monocytes were stimulated with lactate under normoxia, the protein expression of HIF-1α sustained stable during 20 h and was upregulated upon 10 mM lactate (Figure 4A). In the co-cultured and CM cultured THP-1 monocytes, HIF-1α appeared to stable as well in normoxic environment (Figure 4C and 4D). Moreover, the mRNA levels of HIF-1α in THP-1 monocytes stimulated with lactate, or CM, or co-cultured with HCT116 cells using hang culture well, were all increased (Figure 4E). The changes of HIF-1α protein in the models based on other cell lines also presented the consistent results as above (Figure S3). Meanwhile, *in vivo* immunohistochemical staining and tissue immunofluorescence demonstrated that monocytes with high expression of HIF1α were only appeared in colon tissue of CAC group (Figure 4F and 4G).

The stabilization of HIF1α was regulated by its posttranslational hydroxylation via PHDs. Although lactate could increase HIF-1α mRNA transcription, lactate (10 mM) still induced HIF-1α protein expression in the presence of the transcription inhibitor Actinomycin D (5μg/ml) (Figure 4H), suggesting lactate could affect the stabilization of HIF-1α protein. Therefore, we tested whether lactate could interfere with the proline hydroxylase (PHD) reaction, which hydroxylate 2 proline residues of HIF-1α. Considering that PHD activity requires 2-oxoglutarate as a substrate and may therefore be influenced by other carboxylates [20, 21], we designed competition experiments between lactate and 2-oxoglutarate in normoxic THP-1 monocytes. Addition of 2-oxoglutarate to THP-1 monocytes reduced the abundance of HIF-1α induced by lactate in a concentration dependent manner (Figure 4I). Moreover, we detected hydroxylated HIF-1α level to reflect PHD activity. As a result, the level of hydroxylated HIF-1α was decreased in co-cultured, lactate-treated, and CM-stimulated THP-1 monocytes (Figure 4J). PCMB attenuated lactate-induced stabilization and activation of HIF-1α in THP-1 monocytes, resulting in the increase of hydroxylated HIF-1α significantly (Figure 4J).

In summary, the above results suggested that lactate stabilized HIF1α of THP-1 monocytes by inactivating PHDs.

### Lactate promoted DNA-binding activity of HIF1α to promote transcriptions of COX-2 and PEPCK in THP-1 monocytes

**Figure 5:**
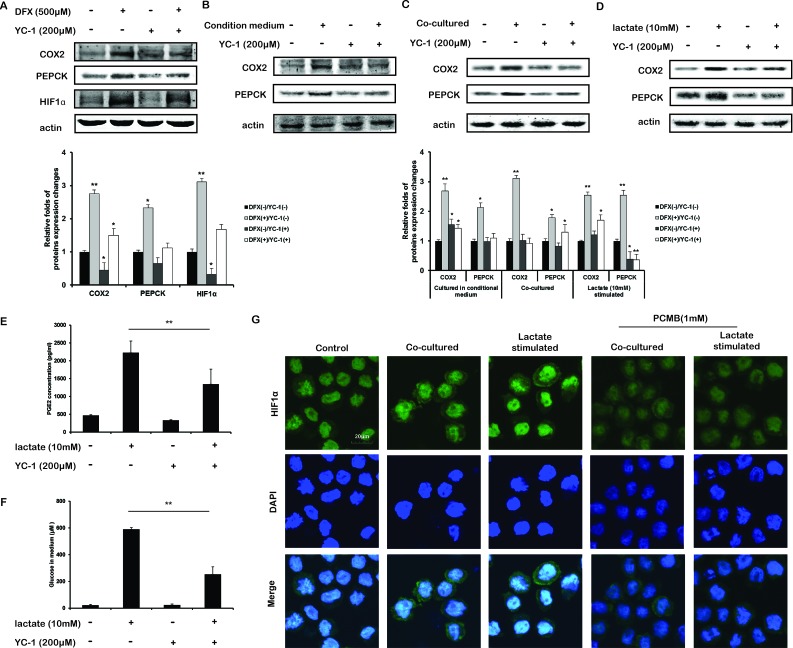
HIF-1α was involved in the lactate-upregulated transcription of COX2 and PEPCK **A.** The effects of DFX (HIF-1α inducer) and YC-1 (HIF-1α inhibitor) on the protein expressions of PECEK and COX2. **B-G.** THP-1 monocytes were cultured condition medium, co-cultured with HCT116 cells, or stimulated with lactate with or without YC-1 for 12 h. **B-D.** The protein expressions of PECEK and COX2 in were measured. Protein bands were quantified. **E, F.** THP-1 monocytes were stimulated with lactate with or without YC-1 for 12 h. PGE2 **E.** and Glucose **F.** in the supernatant were measured. **G.** THP-1 monocytes were stimulated with lactate, or co-cultured with HCT116 cells for 12 h. In the present of or without PCMB, immunofluorescence experiment performed using antibodies specific to HIF-1α and DAPI. Bars, SD; *p < 0.05 or **p < 0.01 versus untreated controls.

Based on the above results, we speculated that whether HIF-1α would regulate PEPCK and COX-2 levels. To examine the transcriptional regulation of HIF-1α on COX2 and PEPCK, THP-1 monocytes were treated with increasing concentrations of the hypoxia surrogate deferoxamine mesylate (DFX, a specific HIF-1α inducer [22]) 500 μM for 12 h. As a result, the DFX treatment stabilized HIF-1α and increased COX-2 and PEPCK protein levels in THP-1 monocytes in normoxia (Figure 5A). Additionally, inhibiting HIF-1α induction using 200 μM YC-1[23] abolished the up-regulation of COX-2 and PEPCK (Figure 5A). In the co-cultured and CM cultured THP-1 monocytes, YC-1 blocked the increase of COX-2 and PEPCK proteins (Figure 5B and 5C). And lactate-increased COX-2 and PEPCK (Figure 5D), as well as PGE2 secretion (Figure 5E) and glucose generation (Figure 5F), were all reversed by YC-1. These results demonstrated that HIF-1α was involved in the upregulation of COX2 and PEPCK in THP-1 monocytes

Then we found the nuclear translocation of HIF-1α were increased in THP-1 monocytes upon lactate stimulation and co-cultured (Figure 5G). The tissue immunofluorescence results also demonstrated a strong nuclear translocation of HIF-1α (Figure 4G, Merge 2). These prompted the function of HIF-1α as a transcription factor. HIF-1α has been shown to bind HRE on promoters of target genes and activate their transcription [24], and COX-2 promoter and PEPCK promoter were both reported to contain a functional HRE [15, 25].

**Figure 6:**
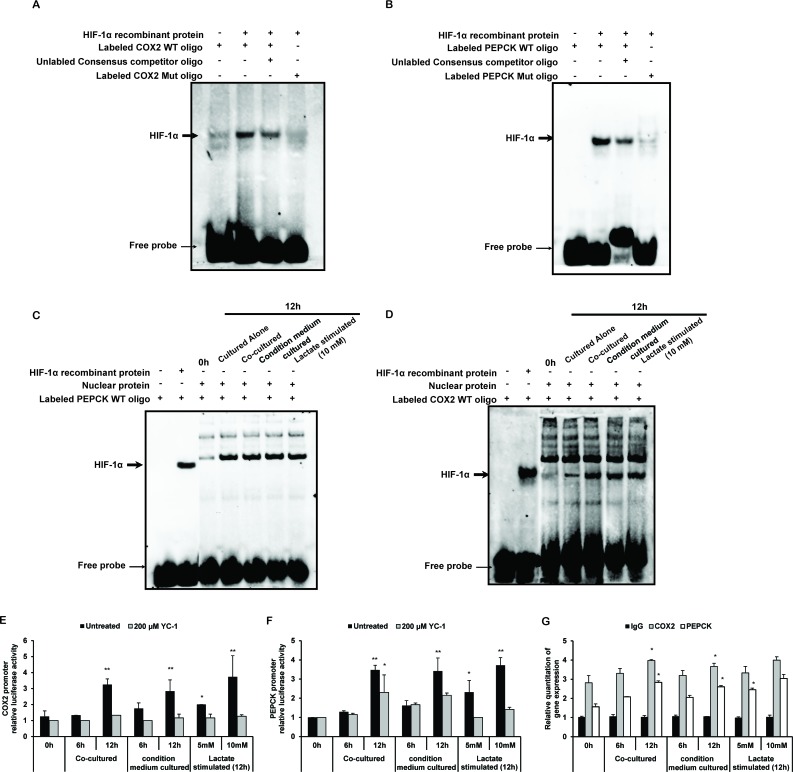
Lactate promotes transcriptions of HIF-1α-targeted genes COX2 and PEPCK **A, B.** Human HIF-1α recombinant protein was subjected to EMSA to assess the binding of HIF-1α to the COX2 promoter and PEPCK promoter. **C, D.** Nuclear were isolated and subjected to EMSA to evaluate the binding of HIF-1α to the COX2 promoter and PEPCK promoter. **E.** In ChIP analysis, DNA was eluted and purified before analysis with specific primers and visualized by quantitative Realtime PCR. **F, G.** COX2 and PECEK promoter luciferase reporter plasmids were transfected into THP-1 monocytes, respectively. Luciferase activity was normalized to Renilla activity and expressed as luciferase/Renilla relative units. Bars, SD; *p < 0.05 or **p < 0.01 versus untreated controls.

To test the physical interaction of HIF-1α with the HRE of the human COX-2 promoter and human PEPCK promoter, we used biotinylated double-stranded oligonucleotides of COX2 and PEPCK containing HRE, respectively, to pull-down HIF-1α. Because HIF-1α might be not the unique transcriptional regulator of COX2 or PRPCK, human HIF-1α recombinant protein was used to indicate the binding site of HIF-1α with COX2 or PRPCK promotor. The bound protein complexes were analyzed by EMSA. As shown in Figures 6A and 6B, HIF-1α bound to the WT HRE oligonucleotide but not to a similar oligonucleotide with the HRE mutated. In the co-cultured, CM cultured, and lactate stimulated THP-1 monocytes, the binding capacity of HIF-1α to the WT oligonucleotide of COX-2 promoter and PEPCK promoter were increased (Figure 6C and 6D). These results indicated that HIF-1α directly bound with the HRE derived from the COX-2 promoter and PEPCK promoter. ChIP analysis showed that HIF-1α could bind to the promoter of COX-2 in co-cultured, CM cultured as well as lactate stimulated THP-1 monocytes endogenously (Figure 6E). Furthermore, to investigate the transcriptional activation of COX2 and PEPCK promoter, we constructed the pGL3-Basic-COX-2-promoter reporter gene plasmid and pGL3-Basic-PEPCK-promoter reporter gene plasmid, which contained the corresponding promoters with wild-type HRE sequence. Lactate promoted the transcriptional activity of COX-2 and PEPCK promoter in THP-1 monocytes, as well as in co-cultured and LS stimulated THP-1 monocytes (Figure 6F and 6G).

All together, these results suggested that COX-2 and PEPCK were the target genes of HIF-1α. Lactate promoted the HIF-1α-regulated positive transcription of COX-2 and PEPCK in THP1 monocytes.

### THP-1 monocytes nourish colorectal cancer HCT116 cells to promote cell growth and glycolysis

In the inflammatory tumor microenvironment, the cancer cells and the inflammatory cells work cooperatively. In the above studies, we found the lactate secreted from colorectal cancer HCT116 cells, could promote the PGE2 and glucose synthesis of THP-1 monocytes. Therefore, we subsequently investigated the influence of THP-1 monocytes on the progression and growth of cancer cells.

**Figure 7:**
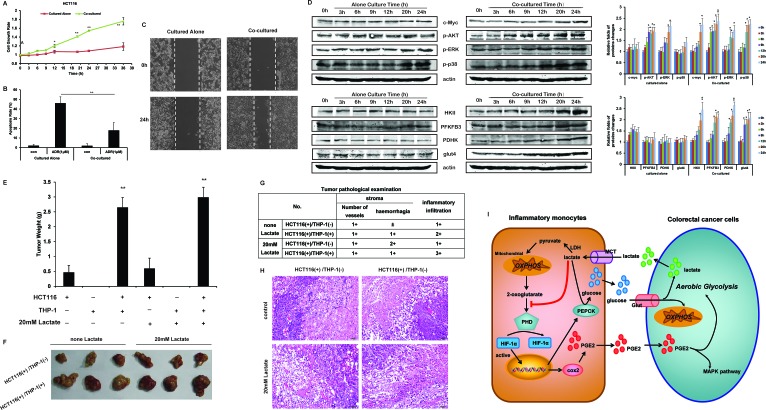
THP-1 monocytes promotes the cell growth of HCT116 cells **A.** HCT116 cell growth rates were measured by MTT assay. **B.** 1 μM ADM treated HCT116 cells were co-cultured with/without THP-1 monocytes for 24 h. The cell apoptosis rates were detected by Annexin V/PI double staining, and quantified. **C.** Cell monolayer was scraped by a sterile micropipette tip. Then HCT116 were co-cultured with/without THP-1 monocytes for 24 h. White lines indicate the wound edge. **D.** HCT116 were cultured alone, or co-cultured with THP-1 monocytes for 24 h. The protein expressions involving with cell growth and glycolysis were detected by Western blot. The actin controls of upper group were the same as those of lower group. Protein bands were quantified. **E.** Heterotopic transplantation tumor animal models was constructed as the describes in the method. The tumor weight were measured. **F.** The photos of the xenograft. **G.** The tumor tissue were assessed by histological analysis. The tags in the table represent the severity : Minor “±”, Mild “1+”, Annual “2+”, Severe “3+”, Extremely severe “4+”, and Normal “-”. **H.** Histological analysis were photographed (×100). **I.** Schematic diagram of lactate driving tumor-inflammation co-evolution. Lactate from cancer cells was taken up by inflammatory monocytes through MCT1. Lactate oxidation increases the intracellular pyruvate, which were available to compete with 2-oxoglutarate from PHD, resulting in HIF-1α protein stabilization and HIF-1 activation. HIF-1α-mediated upregulated transcription of COX2, causing the increased synthesis and secretion of PGE2 in monocytes. Meanwhile, lactate was recycled for gluconeogenesis under the catalysis of PEPCK, which was transcriptional upregulated by HIF-1α as well. In return, inflammatory monocytes fed cancer cells, promoting cell growth of. Bars, SD; **p < 0.01 versus untreated controls.

The capacity of cell growth (Figure 7A), resisting apoptosis (Figure 7B) and mobility (Figure 7C) in HCT116 cells co-cultured with THP-1 monocytes were stronger than the HCT116 cells cultured alone. Moreover, the protein level of c-myc was increased in co-cultured HCT116 cells (Figure 7D). The other significant change was the activation of MAPK pathway, especially ERK and p38. Moreover, the protein levels of key regulators of glycolysis in co-cultured HCT116 cells, including hexokinase II (HKII), pyruvate dehydrogenase kinase (PDHK), lactate dehydrogenase (LDH), glucose transporter 4 (Glut4), and phosphofructokinase 3 (PFBFK3) were all increased (Figure 7D). In further studies, we found the mobility and the capacity resisting apoptosis of HCT116 cells were both strengthened under the treatment of PGE2 (Figure S5A and S5B). And PGE2 activated AKT, ERK, p38 to promote the cell growth (Figure S5C); and upregulated the protein expressions of HKII, Glut4, and PFBFK3 to increased glycolytic capacity of HCT116 cells (Figure S5D). Besides, in other cell lines, including HT29, Caco2, MDA-MB-231 and HepG2 cells, THP-1 monocytes upregulated the expressions of c-myc, p-ERK, HKII, glut4 (Figure S4).

To investigate the effect of inflammatory microenvironment in cancer growth *in vivo*, we developed another *in vivo* model beside CAC model. We mixed THP-1 monocytes and HCT116 cells in 1:1 proportion, and inoculated cell suspension on nude mice. We found that the tumor in HCT116^+^/THP1^+^/lactate^−^ group had significantly strong growth capacity (4.6 folds) than HCT116^+^/THP1^−^/lactate^−^ group (Figure 7E and 7F). However, THP-1 monocytes alone (HCT116 ^−^/THP1^+^/lactate^−^ group and HCT116 ^−^/THP1^+^/lactate^+^ group) had little tumorigenicity (Figure 7E), removing the interference of THP1 monocytes on tumor size and weight. Therefore, these result suggested that THP-1 monocytes promoted the growth of human colon HCT116 cells *in vivo*.

**Figure 8:**
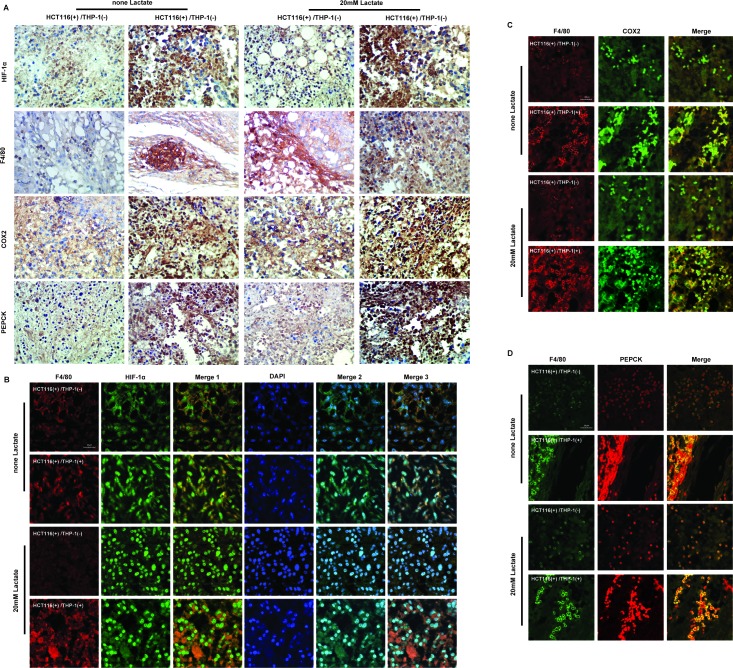
Lactate influences the expressions of HIF-1α, COX2, PEPCK proteins of monocytes in heterotopic transplantation tumor model based on co-culture of HCT116 cells with THP-1 monocytes **A.** Expression level of HIF-1α, COX2, PEPCK and F4/80 in tumor tissue were assessed by immunohistochemistry. (**B**-**D**) Expression level of HIF-1α **B**, COX2 **E** and PEPCK **F** and were detected by IF. The staining of F4/80 was used to distinguish monocytes from the tissue. The figures of Merge 1 were merged by figures marked with F4/80 and HIF-1α; the figures of Merge 2 were merged by figures marked with HIF-1α and DAPI staining; the figures of Merge 3 were merged Merge 1 with and DAPI staining.

Then we added 20 mM lactate to the Matrigel mixing with cell suspension, and inoculated. The tumor weight of the group that contained THP-1 monocytes, the tumor in HCT116^+^/THP1^+^/lactate^+^ group was still bigger HCT116^+^/THP1^−^/lactate^−^ group (Figure 7E). Interestingly, 20 mM exogenous lactate did not showed advantage growth of xenograft in both HCT116^+^/THP1^−^/lactate^+^ group and HCT116^+^/THP1^+^/lactate^+^ group (Figure 7E). However, we analyzed HE staining and found that the HCT116^+^/THP1^+^/lactate^+^ group showed the strongest inflammatory infiltration, and the HCT116^+^/THP1^+^/lactate^−^ group is the next strongest (Figure 7G and 7H). These results demonstrated that lactate did not promote tumor growth directly, but influenced the inflammatory monocytes around cancer cells. Based on this speculation, we detected the expressions of HIF-1α, COX2 and PEPCK in tumor tissue, and marked monocytes by F4/80 (Figure 8). Compared HCT116^−^/THP1^+^/lactate^−^ group with HCT116^+^/THP1^−^/lactate^−^ group, we found the expressions of HIF-1α, COX2 and PEPCK were up-regulated, especially in monocytes. And exogenous lactate could further enhance the levels of HIF-1α, COX2 and PEPCK of THP-1 monocytes.

Taken together, lactate in tumor microenvironment influenced THP-1 monocytes and increased the expressions of HIF-1α, COX2 and PEPCK. Then THP-1 monocytes “fed” colorectal tumor HCT116 cells to promoted tumor growth.

## DISCUSSION

Tumor lactate can predict for metastases and overall survival of patients in several types of human cancers. Although lactate was initially considered merely as an indicator of the glycolysis of normal tissue physiology, many evidences recently transposed to the tumor situation indicate that lactate directly contributes to tumor growth and progression. Here, we modeled the inflammatory tumor microenvironment by co-culture human cancer cells with THP-1 monocytes, and reported a new energy-transfer mechanism of “Two-Compartment Tumor Metabolism” inflammatory tumor microenvironment. As shown in Figure 7I, the lactate from cancer cells is secreted into the microenvironment, and taken up by surrounding THP-1 monocytes through MCT1. In addition to being used as a fuel for oxidative phosphorylation (OXPHOS), lactate brought significant influences to THP-1 monocytes. On one hand, lactate oxidation increases the pool of intracellular pyruvate, which competes with 2-oxoglutarate from PHD, resulting in HIF-1α protein stabilization and HIF-1 activation. Transcription of COX2 is up-regulated, prompting the synthesis of PGE2. On the other hand, lactate in THP-1 monocytes was recycled for gluconeogenesis pathway and synthesize glucose again. The upregulation of PEPCK plays an important role. Both COX2 and PEPCK were targeted genes of HIF-1α. Finally, THP-1 monocytes nourish cancer cells and accelerated tumor growth.

Long before 20^th^ century, lactate was largely considered as a dead-end waste product of glycolysis, resulting in muscle fatigue and acidosis-induced tissue damage. The discovery of Cori Cycle, and emergence of the concept “lactate shuttles” amplified the biological function of lactate [26-28]. These pioneering advances in the lactate activities in normal tissues have only recently been translated into new insight of tumor. Rather than a metabolic intermediate, lactate acts as a signaling molecule and plays significant roles in cancer development. Increasing evidences indicate that lactate in tumors is a fuel for the oxidative metabolism of oxygenated tumor cells [10, 29, 30], a power for the metastasis of tumor and endothelial cells [31, 32], and an important contributor to angiogenesis [33]. Although, the growth-promoting effect of lactate in some certain cancer cells was reported [34], our studies showed that exogenous lactate did not show the direct benefit for tumor growth of HCT116 cells (Figure 7F). We even found that high concentration of exogenous lactate was cytotoxic to tumor (Figure S6). One reason for this result was the significantly down-regulated expression of MCT during the transition from normality to malignancy in human colon [35]. The low expression of MCT1 in colon cancer was beneficial for the development of cancer, and correlated with the deregulation of many butyrate-responsive genes that are involved in the processes of apoptosis, proliferation and differentiation in colon carcinoma tissue. Although lactate could not accelerate tumor growth directly, we found that lactate influenced monocytes in inflammatory tumor microenvironment (Figure 8), promoting the development of cancer. Besides, several studies have shown that lactate influences vascular endothelial cell, tumor-associated fibroblasts, tumor-associated macrophages, and other immune cells to promote tumor angiogenesis, invasion, metastasis and immune escape.

Along with the raise of “tumor microenvironment” conception, THP-1 cell line became an important tool to mimic the inflammatory cells in tumor microenvironment. It has been reported that THP-1 monocytes owned the capability to behave distinct functional programs in response to different microenvironmental signals, such as microbial stimuli or cytokines [36]. In our studies, THP-1 monocytes were affected by the lactate from tumor and then “fed” cancer cells, promoting tumor development. Lactate acted as a hypoxia-mimetic able to activate the transcription factor HIF-1 originally in THP-1 monocytes. The underlying pathway required lactate oxidation into pyruvate to compete with 2-oxoglutarate (a by-product of the TCA cycle) for the control of PHD activity. Activated HIF-1α in THP-1 monocytes increased the transcription of COX2, promoting PGE2 synthesis. In addition to PGE2, human monocytes “fed” cancer cells with high-energy fuel glucose, which was generated by the recycle of lactate. The transcriptional upregulation of rate-limiting enzyme (PEPCK, FBPase, and G6Pase) in gluconeogenesis were responsible for the glucose generation. Beside the HIF-1α-mediated transcription of PEPCK, we found that the protein level of FBPase was upregulated in co-cultured condition (Figure 3D). The induction of HIF-1α by DFX and the inhibition of HIF-1α by YC-1 had little influence on FBPase expression (Figure S7A and S7B), suggesting that FBPase was not the targeted gene of HIF-1α. Therefore, some other transcription factor, including peroxisome proliferator activated receptors (PPARs), PPARγ coactivator 1α (PGC1α), and estrogen-related receptors (ERRs), were investigated. PPARs modulate the expression of genes regulating glucose and lipid metabolism, forced glycolysis-dependent cancer cells into “metabolic catastrophe” [37]. PGC1α is a transcription coactivator that interacts with several nuclear receptors known to bind gluconeogenic promoters, such as PPARs [38]. The degradation of PGC1α down-regulated the gene expressions of PEPCK and G6Pase [39]. And ERRs and PGC1α work in concert to regulate mitochondrial biogenesis and metabolic pathways [40, 41]. We found that the cotransfection of PGC1α and ERRα increased the expressions of FBPase and PEPCK; and the cotransfection PGC1α with PPARγ upregulated FBPase as well (Figure S7D and S7E). Although there was little influence on the expressions of PPARγ, PGC1α and ERRα. In THP-1 monocytes co-cultured with HCT116 cells (Figure S7C), lactate could bind with PPARγ and affect it directly. Through molecular docking study, we found lactate could not bind with PGC1α, and had quite low binding activity with ERRα, but had a high and stable affinity with the binding site between PGC1α and PPARγ (Figure S7F). All these results suggested that HIF-1α was not the unique factor affected by lactate in tumor microenvironment. The search for the factors that be modulated by lactate will provide infinite possibilities for future cancer therapeutic approaches.

Lactate export and uptake is a MCT-dependent process. The normoxic cells with oxidative potential have the preference to express MCT 1; instead glycolytic/hypoxic cells favor MCT4. Accordingly, MCT4 facilitates the release of lactate from glycolytic cells [42, 43], and MCT1 could primarily mediate lactate uptake by oxygenated cells in tumors [8, 44]. Here, we found the lactate uptake in THP-1 monocytes was dependent on MCT1. So far, no specific small molecule inhibitor or blocking antibody selective for MCT has been identified. Due to the significance of lactate in cancer progression, therapeutic agents targeting MCT1could exert multiple anticancer influences. A first MCT1 inhibitor, AZD3965, is currently entering Phase I/II clinical trials for advanced solid tumors [7] (http://science.cancerresearchuk.org/). Although many of the biological activities of lactic acid in tumors could be targeted therapeutically with MCT inhibitors, most intrinsic activities of the lactate anion remain to be identified, characterized and, potentially, tailored for therapy. Recently, mTOR inhibitor rapamycin was reported as a pharmacological inhibitor of lactate generation in cancer cells [45]. Rapamycin decreases lactate production independent of respiration by suppressing mTOR-mediated activation of HIF-1α [46]. Over production of lactate by tumors can lead to fatal lactic acidosis in cancer patients [47]. Therefore, rapamycin may be used to treat lactic acidosis both in cancer patients. More importantly, over produced lactate stromal cells or tumor cells could be inhibited by rapamycin, resulting in the fail of energy-transfer mechanism of feeding cancer cells. The modulation of lactate in tumor microenvironment provides a new insight for cancer therapy.

Tumor cells perform a metabolic switch to produce intermediates for increased cell growth and division. This appears to be a very early event in carcinogenesis, at least in a significant number of cases observed so far. On the basis of our current knowledge, it is too early to draw firm conclusions about a causative role of deregulated glycolysis in tumorigenesis. Here, we demonstrated the energy-transfer mechanism of cancer metabolism in inflammatory tumor microenvironment. THP-1 monocytes recycle lactate to feed cancer cells with glucose and PGE2 in favor of cancer progression. This studies provide a potential relationship between metabolic disorder and inflammatory. Trying to elucidate the “hen and egg problem” in the sequence of inflammatory events, metabolic deregulation, genetic alterations, and acquisition of functional malignancy represents an exciting challenge in experimental cancer research.

## MATERIALS AND METHODS

### Reagents

Lactate solution, deferoxamine mesylate (DFX, HIF-1α inducer), YC-1 (3-(5′-Hydroxymethyl-2′-furyl)-1-benzyl indazole, HIF-1α inhibitor), dactinomycin D and PGE2 were purchased from Sigma-Aldrich (Sigma-Aldrich, St. Louis, MO, USA). 4-(Chloromercuric) benzoic acid (PCMB) and 2-oxoglutarate were purchased from Aladdin (Aladdin-reagent Database Inc., Shanghai, China).

### Cell culture

HCT116 cells and THP-1 monocytes were respectively cultured in McCoy's 5A medium (Sigma, St Louis, MN) and RPMI-1640 medium (Gibco, Carlsbad, CA), supplemented with 10% fetal bovine serum (Gibco, Carlsbad, CA), 100 U/mL benzyl penicillin and 100 μg/mL streptomycin. Cells were cultured in a humidiﬁed environment with 5% CO_2_ at 37°C.

For conditional culture of human THP-1 monocytes, HCT116 cells were cultured as above for 48 h. Then the condition medium (culture supernatants) were collected and used to stimulate THP-1 monocytes for different times (3, 6, 9, 12, 20, 24 h). THP-1 monocytes were cultured alone as controls.

For co-culture of human colon cancer HCT116 cells with human THP-1 monocytes, HCT116 cells were seeded in 24-well plates (Costar, Cambridge, MA) at a density of 4×10^4^ cells per well and grown to 80% confluence. THP-1 monocytes were collected by centrifugation (600 g for 10 min), washed, and added to the HCT116 cell culture at 10:1 for 24 h using Milli hang culture well. THP-1 monocytes were cultured alone as controls.

### Lactic acid production

To determine the generation of lactic acid, medium were collected and assayed following the manufacturer's instructions of the Lactic Acid production Detection kit (KeyGen, Nanjing, China). The assay was detected using a spectrophotometer (Thermo, Waltham, MA, USA) at 570 nm.

### Glucose uptake assay

For the analysis of glucose uptake, the Amplex Red Glucose Assay Kit (Invitrogen, Eugene, OR) was used. Culture medium was collected and diluted 1:4000 in water. The amount of glucose in the medium was then detected using the Amplex Red Assay according to the manufacturer's instructions. The detection was performed by spectrophotometer (Thermo, Waltham, MA) at Ex/Em = 530 nm/590 nm.

### PGE2 Quantification

PGE2 level was determined in growth medium using PGE2 ELISA kit (KeyGen, Nanjing, China) following the manufacturer's instructions. Levels of cytokines were expressed in pg/mL.

### Western blotting

Proteins were isolated using lysis buffer, incubated in SDS buffer, separated on SDS-polyacrylamide gels, and electroblotted onto PVDF membranes. Immunoreactive protein bands were detected using an Odyssey Scanning System (LI-COR Inc., Superior St., Lincoln, NE, USA). The following antibodies were used for Western blotting: G6Pase, FBPase, PEPCK, COX2, c-Myc, p-AKT, p-p38, PDHK, MCT1, β-actin (Santa Cruz, CA, USA) at 1:400 dilution; MCT4, p-ERK, GLUT 4, (Bioworld Technology, Inc., MN) at 1:800 dilution; HIF-1α, Hexokinase II, Hydroxy-HIF-1α, PFKFB 3 (Cell Signaling Technology, Inc., MA) at 1:800 dilution.

### Real-time PCR analysis

Total RNA was extracted using the TriPure Isolation Reagent (Roche Diagnostics, Mannheim, Germany), and then amplified by polymerase chain reaction (PCR). The primer sets used in the PCR amplification were as follows:

PEPCK-sense (5′-AAGAGACACAGTGCCCATCC-3′),
PEPCK-antisense (5′-ACGTAGGGTGAATCCGTCAG-3′),
COX2-sense (5′-ATAAGCGAGGGCCAGCTTTCA-3′),
COX2-antisense (5′-GTGGGAGGA TACATCTCTCCA-3′),
HIF-1α-sense (5′-CAGCCGCTGGAGACACAATC-3′),
HIF-1α-antisense (5′-TTTCAGCGGTGGGTAATGGA-3′),
β-actin-sense (5′-TCCTTCCTGGGCATGGAGTC-3′),
β-actin-antisense (5′-TTCTGCATCCTGTCGGCAATG-3′).

The relative gene expressions were analyzed using quantitative RT-PCR with β-actin as an internal control.

### Immunofluorescence confocal microscopy

Cells were fixed with 4% paraformaldehyde in PBS at 20 min intervals, permeabilized with 0.5% Triton X-100, and blocked with 3% BSA for 30 min. Tissue sections were heat fixed, deparaffinized and rehydrated through a graded alcohol series (100%, 95%, 85%, 75%) to distilled water. Tissue sections were boiled in citrate buffer at high temperature for antigen retrieval, and treated with 3% hydrogen peroxide to block endogenous peroxidase activity. The slides were incubated with a protein-blocking agent (kit 9710 MAIXIN, Maixin-Bio Co., Fujian, China) prior to the application of the primary antibody.

Incubation with primary antibodies was done overnight at 4°C. Then the nuclei were stained with 4′,6-diamidino-2-phenylindole (DAPI, Sigma-Aldrich, St. Louis, Mo) 20 minutes before imaging. A laser scanning confocal microscope FV10-ASW [Ver 2.1] (Olympus Corp, MPE FV1000) was used for co-localization analysis.

### Cell transfection and luciferase reporter assay

The transcriptional activities of HIF-1α to COX2 or PEPCK were analyzed by luciferase reporter gene assays. pGL3-Basic-COX2-luc plasmid and pGL3-Basic-PEPCK-luc plasmid (Beyotime, Nantong, China) containing COX2 or PEPCK binding motifs (Sequences of the plasmids were listed in Figure S3A and S3B) using the Lipofectamine 2000TM reagent (Invitrogen, CA, USA) according to the manufacturer's instructions. The luciferase reporter gene plasmids were added to equalize the total amount of DNA (4 μg/well in a 6-well plate) while the Renilla luciferase reporter was used at 0.4 μg/well in a 6-well plate and served as the normalization control. After 12 h, luciferase assays were performed with the Luciferase Reporter Gene Assay kit (Promega, Madison, WI, USA) and detected using Luminoskan ascent (Thermo, Waltham, MA, USA).

### Chromatin immunoprecipitation (ChIP) assay

ChIP assay was performed following previously published protocols with some modifications. Cells were cross-linked with formaldehyde, quenched with glycine, sonicated on ice and centrifuged at 4°C. Mixtures were incubated with anti-COX2 or anti-PEPCK or pre-immune IgG with rotation at 4°C overnight and then incubated with Protein A/G Agarose at 4°C for 6 h. Finally, immune complexes were captured by Protein A/G Agarose and eluted with elution buffer (1% SDS, 0.1 M NaHCO_3_) at 37°C for 30 min. Cross-linking was reversed at 65°C for 4 h in a high salt buffer (0.2 M NaCl, 50 mM Tris pH 6.5, 10 mM EDTA, 0.2 mg/ml proteinase K). Extracted and dissolved immunoprecipitated DNA was quantified by real-time PCR with primers encompassing the COX2 or PEPCK binding sites. Primers for COX2 or PEPCK promoter quantification were listed in Figure S8C. An equal volume of non-precipitated (input) genomic DNA was used to correct for the differences in PCR amplification efficiencies and amounts of DNA. The PCR analyses were performed using the Real-time PCR kit (TaKaRa Biotechnology Co. Ltd., Dalian, China).

### Electrophoretic mobility shift assay (EMSA)

EMSA was performed using the Chemiluminescent EMSA Kit (Beyotime, Nantong, China) following the manufacturer's protocol. Briefly, human HIF-1α recombinant protein (0.8 μg/sample, Abcam Ltd, HK, China) or nuclear extracts (8 μg/sample) were incubated with biotin-labeled oligonucleotides and biotin-labeled mutant oligonucleotides (Sequences of the oligonucleotides were listed in Figure S8D) in reaction buffers, for 30 min at room temperature. DNA-protein complexes were separated from free oligonucleotides on 6% native polyacrylamide gels. The gels were visualized with the Bio-Rad Infrared system and quantified using Image LabTM software (Bio-Rad, Berkeley, CA, USA).

### Colitis and colitis-associated cancer animal models

Female and male C57BL/6 mice, 35-40 days old, weighing 18-22 g, were supplied by Shanghai Laboratory Animal Center, China Academy of Sciences (Certificate No. 122). The mice were raised in air-conditioned rooms under controlled lighting (12 h light/day) and provided with food and water at discretion. Animal care and surgery protocols were approved by the Animal Care Committee of China Pharmaceutical University. All animals were treated and used in a scientifically valid and ethical manner. A total of 30 mice (male:female = 1:1) were randomly divided into the following 3 groups (10 mice /group): normal (negative control group), DSS (Colitis group), and AOM/DSS (azoxymethane-based colitis-associated cancer, CAC group).

In the acute colitis model, mice were given 3.5% DSS in drinking water for 7 days. DSS-treated groups received 0.9% normal saline in a comparable volume by the same route. Normal control mice received filtered water alone. Mice were sacrificed on Day 7 and clinical parameters and pathology were evaluated.

Colitis-associated cancer (CAC) was induced as described previously [48]. Briefly, on day 1, mice in carcinoma group were injected intraperitoneally (i.p.) with 12.5 mg/kg AOM (azoxymethane, Sigma, St Louis, MN) and maintained on a regular diet and water for 5 days. After 5 days, mice received 2.5% DSS (dextran sulfate sodium, MP Biomedicals Inc., Irvine, CA) in drinking water for 5 days. After this, mice were maintained on regular water for 14 days and subjected to two more DSS treatment cycles. Body weight was measured every week. On day 95, mice were sacrificed.

In both the acute colitis and the CAC model the mice were examined daily for behavior, water/food consumption, body weight, stool consistency, and the presence of gross blood in the stool or at the anus.

### Heterotopic transplantation tumor animal models

This experiment was conducted in accordance with the guidelines issued by the State Food and Drug Administration (SFDA of China). Female athymic BALB/c nude mice (35–40 days old) with body weight ranging from 18 to 22 g were supplied by the Academy of Military Medical Sciences of the Chinese People's Liberation Army (Certificate No. SCXK-(Army) 2007-004). Forty nude mice were randomly assigned to six groups. Three of the groups were respectively injected with Matrigel plug containing 100 μl 10^6^ HCT116 cells suspension alone, or 100 μl 10^6^ THP1 monocytes suspension alone, or 200 μl suspension mixed with HCT116 and THP1 cells (1:1), and 20 mM lactate. The other three groups respectively injected with Matrigel plug containing 100 μl 10^6^ HCT116 cells suspension alone, or 100 μl 10^6^ THP1 monocytes suspension alone, or 200 μl suspension mixed with HCT116 and THP1 cells (1:1), and lactate was replaced by an equal volume of saline. Tumor growth was determined every 3 days. Longest and shortest diameters were measured with an electronic caliper and the formula of a prolate ellipsoid was used to calculate the volume of the tumor. On day 21, mice were sacrificed. The plugs were dissected and used for histological analysis and immunohistochemistry assay as described below.

### Histological analysis and immunohistochemistry

Four-micronthick sections were prepared from formalin-fixed, paraffin-embedded colon tissue from model mice and stained with H&E. Immunohistochemical staining against HIF-1α, COX2 and PEPCK was performed with standard techniques. Image pro plus software was used to analyze the number of positive cells by detecting IOD.

### Cell growth inhibition and apoptosis assay

The cell growth inhibition and apoptosis effect were evaluated following previously published protocols with some modifications by Colorimetric MTT-Assay and Annexin-V/PI double-staining assay, respectively [49].

### Wound healing assay

The wound healing assay is used to study directional cell migration *in vitro*. HCT116 cells were seeded in a six-well plate and allowed to attach overnight to 80% confluency. Subsequently, cell monolayers were wounded by white pipette tips and washed with PBS twice to remove floating cells. Cells were then co-cultured with THP-1 monocytes or incubated in 200 nM PGE2 for 24 h. Cells migrated into the wound surface and the number of migrating cells was determined under an inverted microscopy. Five randomly chosen fields were analyzed for each well. The percentage of inhibition was expressed using untreated wells at 100%.

### Molecular docking

We used our protein-ligand docking software package GOLD to dock lactate molecular into estrogen-related receptor alpha (ERRα) or the bind site of peroxisome proliferator-activated receptor gamma (PPAR-γ) with peroxisome proliferator-activated receptor gamma coactivator 1-alpha (PGC1α), respectively. Molecular docking was performed following the method of Sun et al. (2010). We then ranked each protein according to the GOLD fitness score. Molecular interactions were observed using Discovery Studio (Accelrys, San Diego, CA).

### Statistical evaluation

Data are presented as mean ± SD from triplicate parallel experiments unless otherwise indicated. Statistical analyses were performed using one-way ANOVA.

## SUPPLEMENTARY MATERIAL FIGURES


