# Supplementary material for: Lactate promotes PGE2 synthesis and gluconeogenesis in monocytes to benefit the growth of inflammation-associated colorectal tumor
Source: Oncotarget. 2015 Apr 15;6(18):16198–214. doi: 10.18632/oncotarget.3838 (PMC4594635; doi:10.18632/oncotarget.3838)
Supplement: Supplementary file 1 [file oncotarget-06-16198-s001.pdf]

# Lactate promotes PGE2 synthesis and gluconeogenesis in monocytes to benefit the growth of inflammation-associated colorectal tumor

## Supplementary Material

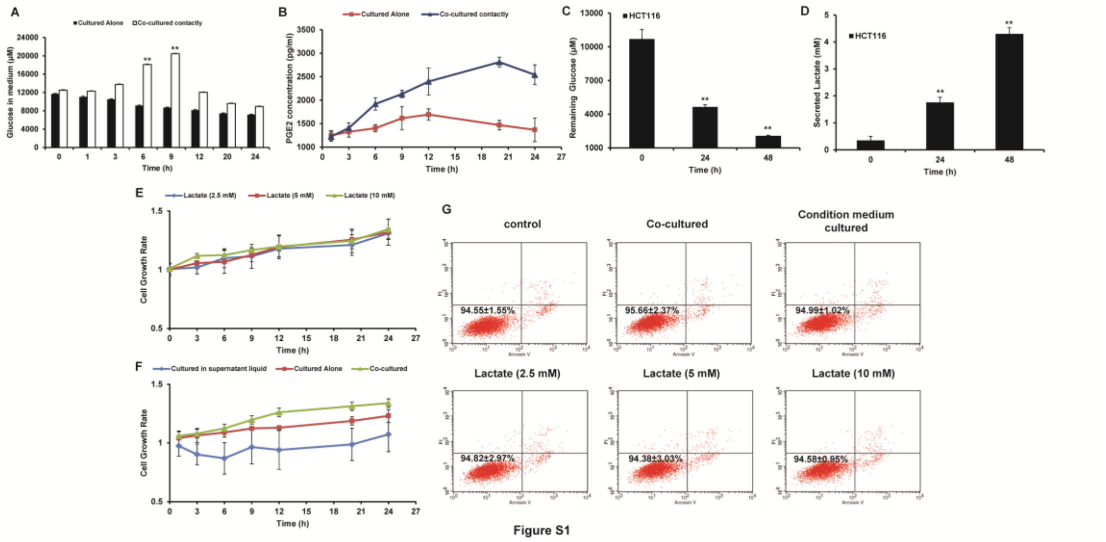


Figure S1

**Figure S1:** (A, B) THP-1 monocytes were co-cultured with HCT116 cells contact for 24 h. Of different time points, the quantities of glucose (A) and PGE2 (B) in the supernatant were measured by Amplex Red assay kit and PGE2 ELISA kit, respectively. (C, D) HCT116 cells were cultured alone for 48 h. Lactate (C), and glucose (D) in the supernatant were measured. (E) The cell viability of THP-1 monocytes treated with increasing concentrations of lactate was measured by MTT assay. (F) The cell viability of THP-1 monocytes or cultured alone, cultured in CM , and co-cultured with HCT116 cells were measured by MTT assay. (G) Growth of THP-1 monocytes were detected by Annexin V/PI double staining assay. Bars, SD; *$p<0.05$ or **$p<0.01$ versus untreated controls.

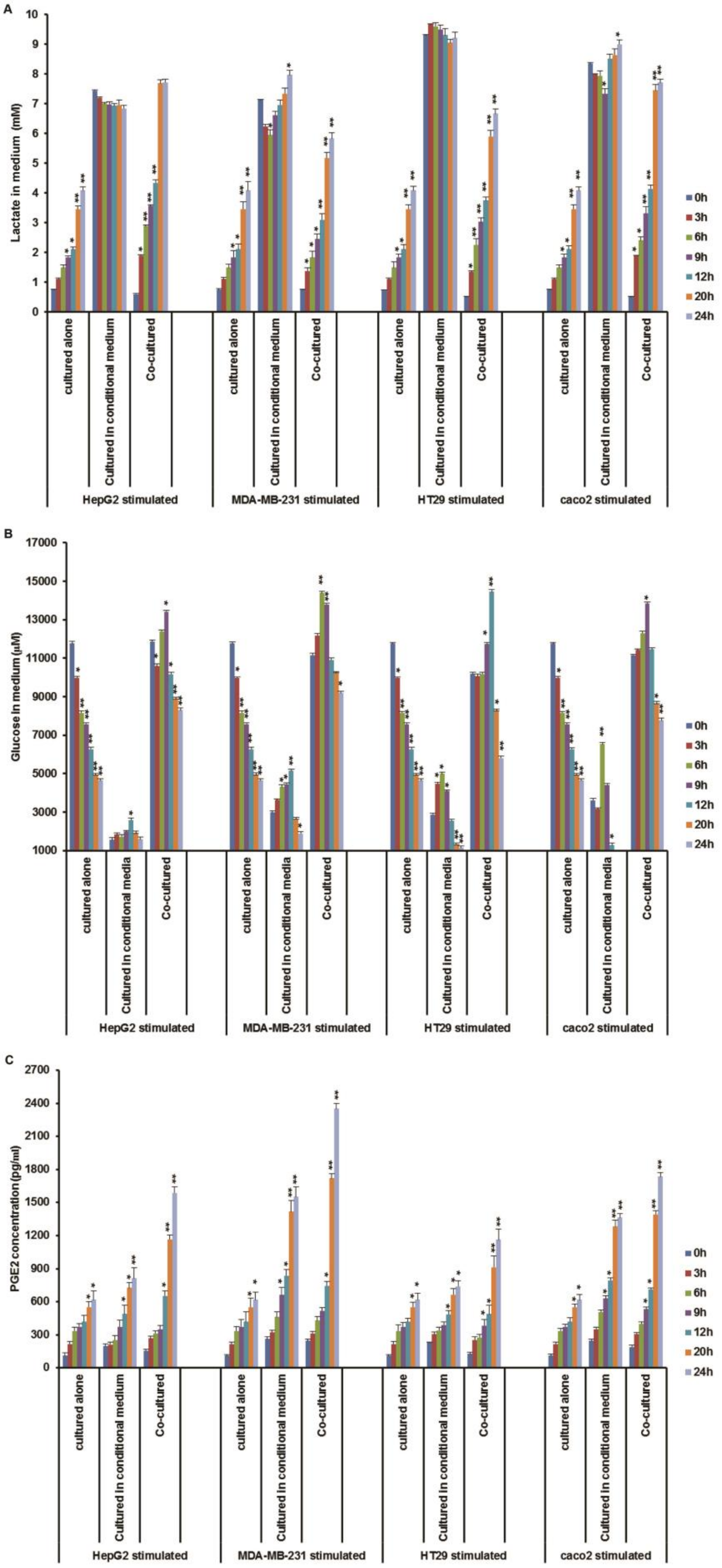
A
Lactate in medium (mM)
cultured alone
Cultured in conditional medium
Co-cultured
HepG2 stimulated
MDA-MB-231 stimulated
HT29 stimulated
caco2 stimulated
0h
3h
6h
9h
12h
20h
24h
B
Glucose in medium (μM)
Cultured in conditional media
C
PGE2 concentration (pg/ml)

Figure S2

**Figure S2: The changes of glucose metabolism and PGE2 secretion of THP-1 monocyte based on other cancer cell lines.** THP-1 monocytes were stimulated by CM of HepG2 cells, MDA-MB-231 cells, HT29 cells and Caco2 cells, or co-cultured with them, respectively for 24 h. (A) The quantities of lactate (B),  in the supernatant were measured by Lactic Acid production Detection kit. (B) The quantities of glucose in the supernatant were measured by Amplex Red assay kit. (C) The quantities of PGE2 in the supernatant were measured by PGE2 ELISA kit. The models based on the four cancer cell lines used the same control of THP1 cultured alone. Bars, SD; $^{*}p<0.05$ or $^{**}p<0.01$ versus untreated controls.

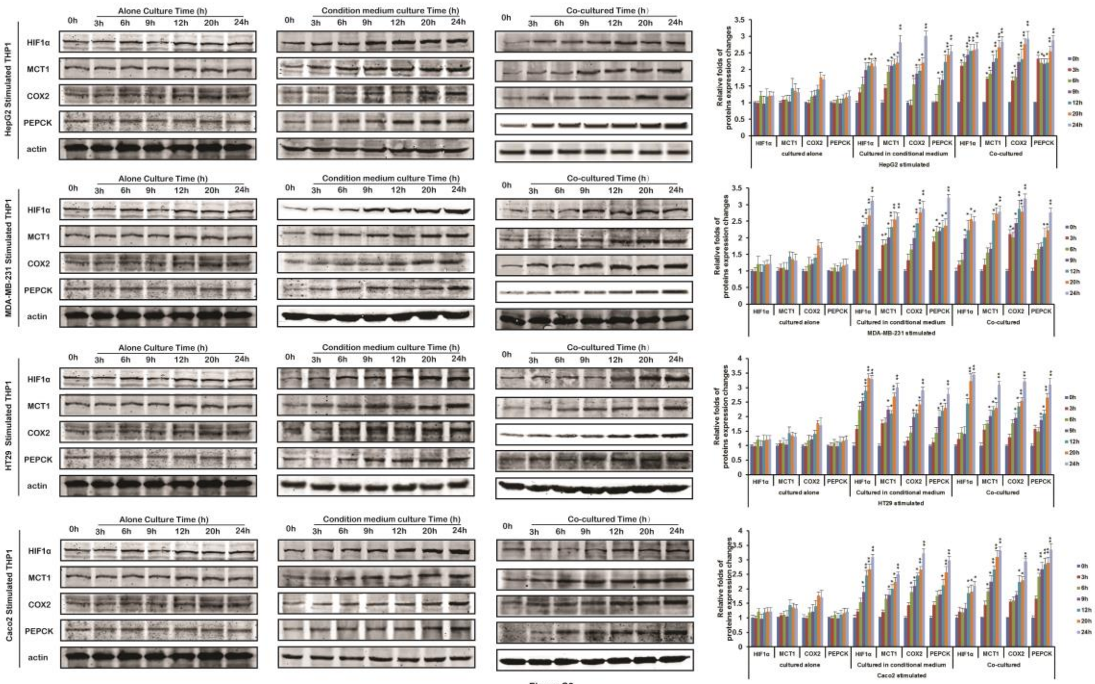


Figure S3

**Figure S3: The changes of MCT1, HIF-1α, COX2 and PEPCK protein expressions in THP-1 monocytes based on other cancer cell lines.** THP-1 monocytes were stimulated by CM of HepG2 cells, MDA-MB-231 cells, HT29 cells and Caco2 cells, or co-cultured with them, respectively for 24 h. Then protein expressions of MCT1, HIF-1α, COX2 and PEPCK were detected by western blot. The models based on the four cancer cell lines used the same control of THP1 cultured alone. Protein bands were quantified. Bars, SD; *p<0.05 or **p<0.01 versus untreated controls.

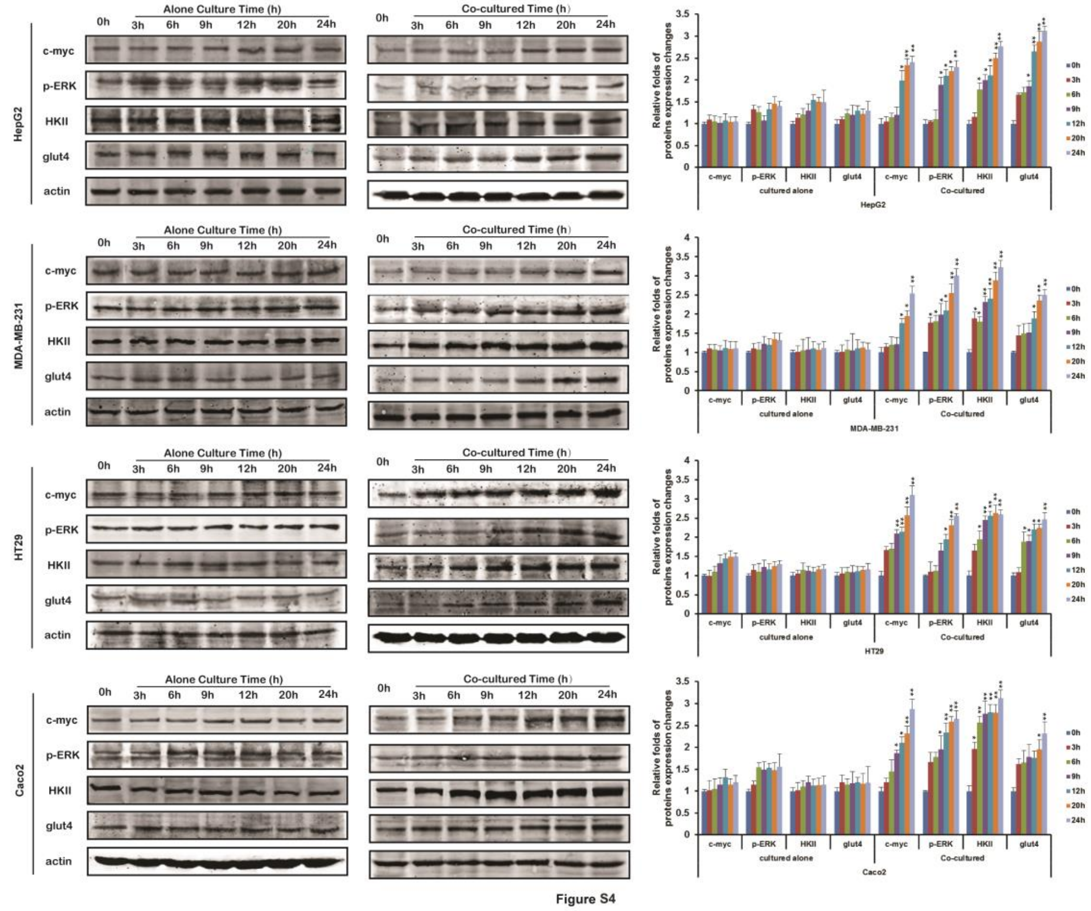


**Figure S4: The influences of THP-1 monocytes on the protein expressions involving with cell growth and glycolysis in cancer cells.** HepG2 cells, MDA-MB-231 cells, HT29 cells and Caco2 cells were cultured alone, or co-cultured with THP-1 monocytes for 24 h. The protein expressions of c-myc, p-ERK, HKII and glut4 were detected by Western blot. Protein bands were quantified. Bars, SD; *$p<0.05$ or **$p<0.01$ versus untreated controls.

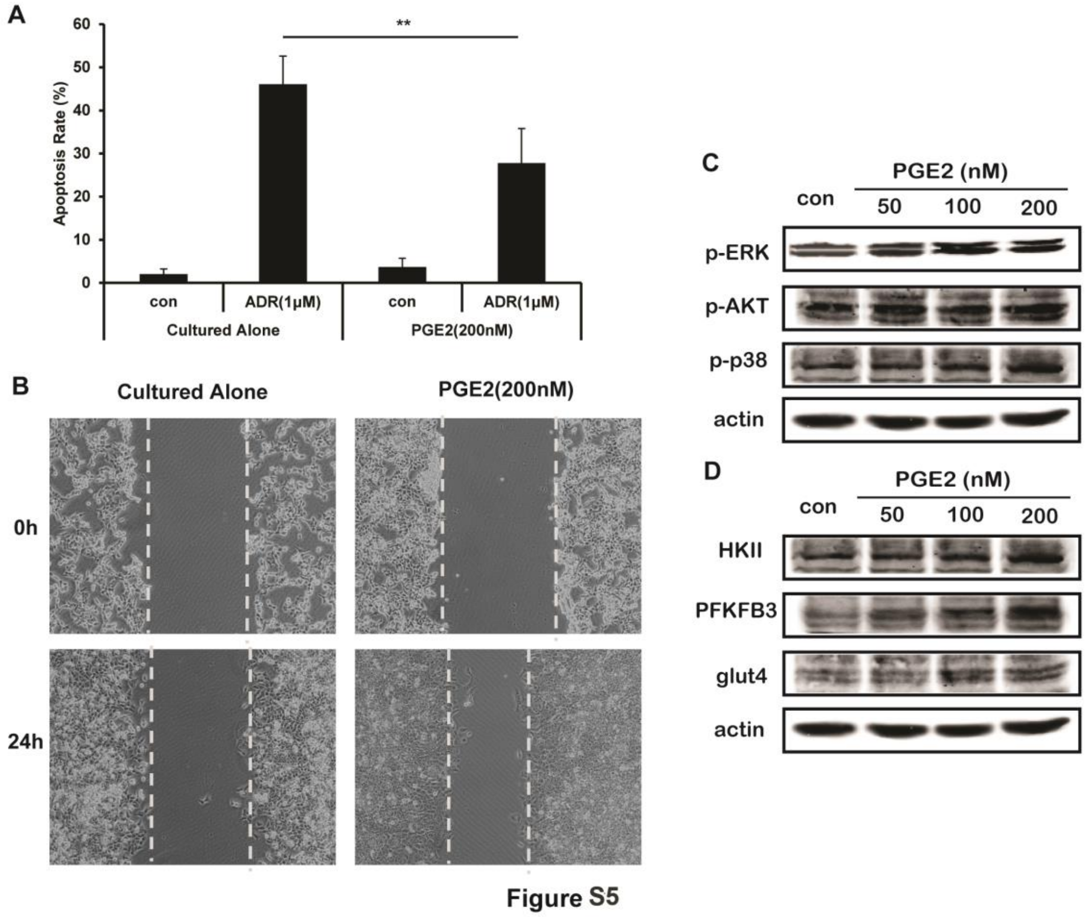


**Figure S5: PGE2 promoted the cell growth of HCT116 cells.** (A) 1 μM ADM treated HCT116 cells were treated with 200 nM PGE2 for 24 h. The cell apoptosis rates were detected by Annexin V/PI double staining, and quantified. (B) Cell monolayer was scraped by a sterile micropipette tip. Then HCT116 were treated with 200 nM PGE2 for 24 h. White lines indicate the wound edge. (C, D) HCT116 cells were treated with PGE2 for 24 h. The protein expressions involving with cell growth (C) and glycolysis (D) were detected by Western blot. The controls of Figure S5C and S5D were the same. Because the actual data were from the same experimental dataset. Bars, SD; *$p<0.05$ or **$p<0.01$ versus untreated controls.

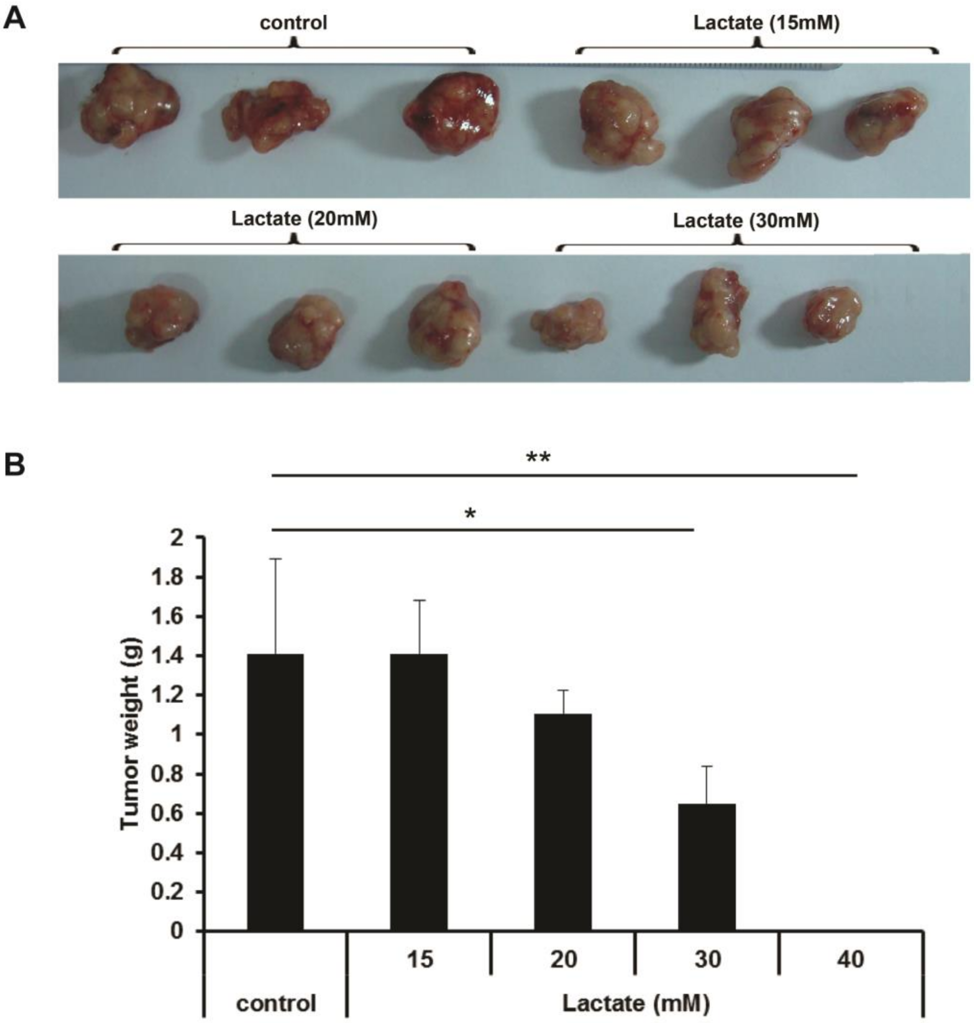


Figure S6

**Figure S6: The direct effect of lactate on tumor growth.** Nude mice were inoculate inoculated subcutaneously with 1 × $10^6$/100μl HCT116 cells into the right axilla.(A) The tumor weight were measured. (B) The photos of the xenograft. Bars, SD; *p<0.05 or **p<0.01 versus untreated controls.

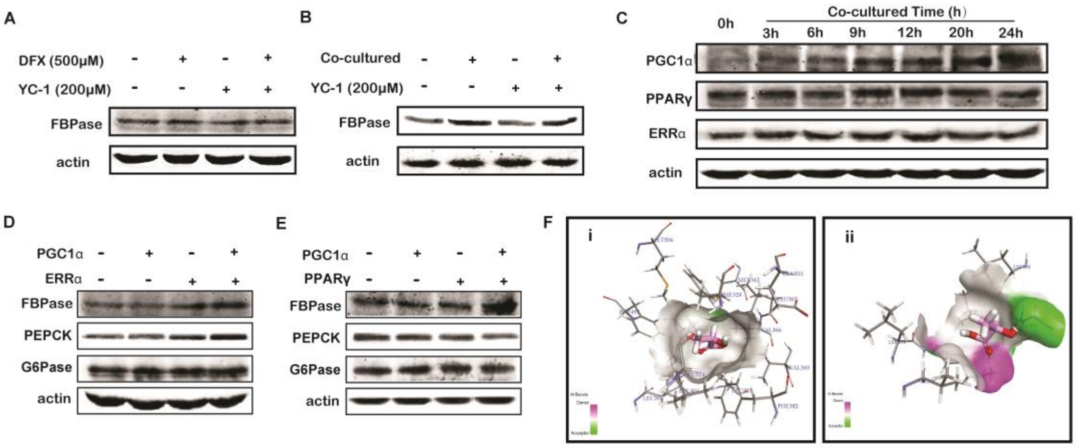


Figure S7

**Figure S7:** (A) The protein expression of FBPase in the THP-1 monocytes that were incubated with 200 μM DFX and/or 200 μM YC-1,were detected by Western blot. (B) The protein expression of FBPase in the THP-1 monocytes, which were co-cultured with HCT116 cells and incubated with/without 200 μM YC-1,were detected by Western blot. (C) In different time points, the protein expression of PPARγ, PGC1α and ERRα in THP-1 monocytes co-cultured with HCT116 cells for 24 h were assayed by Western blot. (D) THP-1 monocytes were transfected with PGC1α and/or ERRα plasmids (Addgene, MA), then the protein expressions of PEPCK, FBPase, and G6Pase were detected by Western blot. (E) THP-1 monocytes were transfected with PGC1α and/or PPARγ plasmids (Addgene, MA), then the protein expressions of PEPCK, FBPase, and G6Pase were detected by Western Blot. (F) Binding mode of the lactate from virtual screening with ERRα (i), and the binding site between PPARγ and PGC1α (ii).

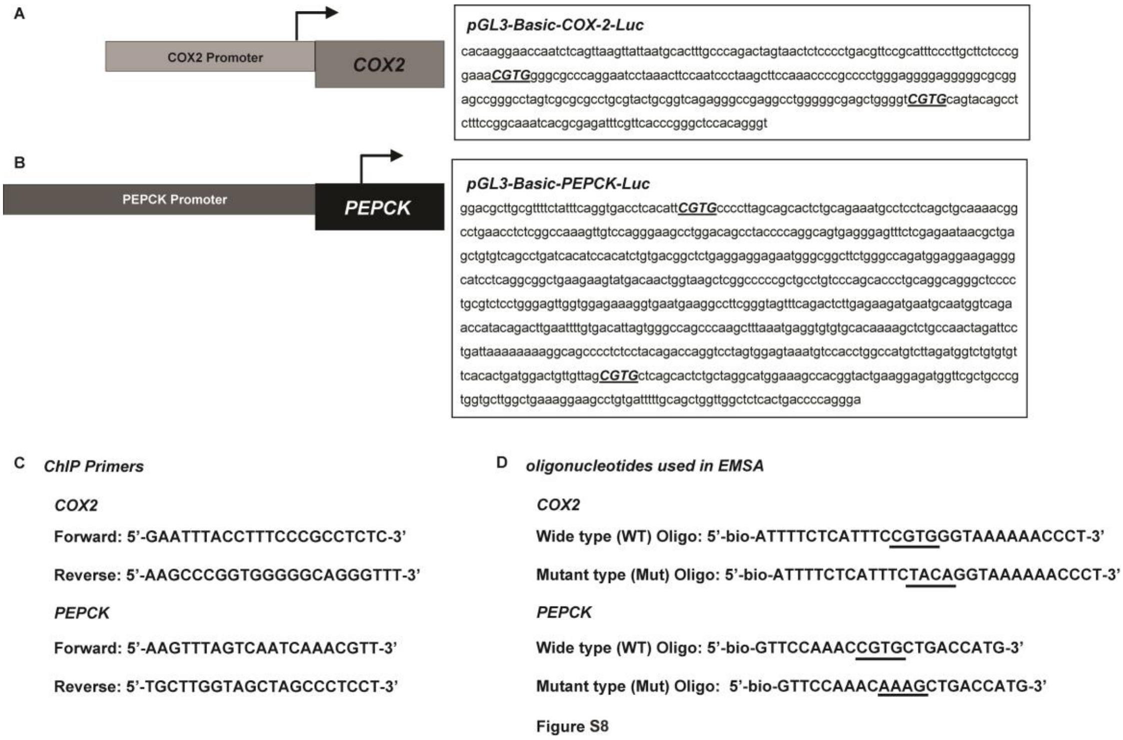


**Figure S8:** (A, B) The nucleotide sequence of pGL3-Basic-COX2-luc plasmid (A) and pGL3-Basic-PEPCK-luc plasmid (B). COX2 and PEPEK contain HIF-1α DNA-binding sites in the upstream regulatory promoter regions. The 5’-upstream regulatory regions were analyzed for potential HIF-1α cis-acting DNA binding / enhancer sites using software available at Entrez Genome and the Genetics Computer Group. (C) Primers for COX2 or PEPCK promoter quantification in CHIP assay. (D) Biotin-labeled oligonucleotides and biotin-labeled mutant oligonucleotides of COX2 and PEPCK for EMSA assay.
